# Altered perivascular-aligned diffusivity in postherpetic neuralgia assessed by the DTI-ALPS index

**DOI:** 10.3389/fnins.2026.1814723

**Published:** 2026-05-19

**Authors:** Daeseok Oh, Dong Ah Lee, Ho-Joon Lee, Hyun-Seong Lee, Kang Min Park

**Affiliations:** 1 Department of Anesthesiology and Pain Medicine, Haeundae Paik Hospital, Inje University College of Medicine, Busan, Republic of Korea; 2 Department of Neurology, Haeundae Paik Hospital, Inje University College of Medicine, Busan, Republic of Korea; 3 Department of Radiology, Haeundae Paik Hospital, Inje University College of Medicine, Busan, Republic of Korea

**Keywords:** diffusion tensor imaging, DTI-ALPS, glymphatic system, pain, postherpetic neuralgia

## Abstract

**Background:**

The glymphatic system has been proposed as a key pathway for interstitial fluid clearance in the brain, but its role in chronic neuropathic pain conditions such as postherpetic neuralgia (PHN) remains unclear. The diffusion tensor image analysis along the perivascular space (DTI-ALPS) index has been suggested as a noninvasive proxy of perivascular-aligned diffusivity related to glymphatic-associated processes.

**Methods:**

A total of 83 participants were recruited, comprising 42 patients with PHN and 41 age- and sex-matched healthy controls. All participants underwent DTI, and the DTI-ALPS index was calculated using DSI Studio. The primary group comparison was performed using analysis of covariance adjusted for age and white matter hypointensity volume. In the PHN group, neuropathic symptom severity was assessed using the Douleur Neuropathique en 4 questionnaire (DN4).

**Results:**

In the primary adjusted analysis, the DTI-ALPS index remained lower in patients with PHN than in healthy controls (estimated marginal means: 1.247 vs. 1.348; mean difference = −0.100, 95% CI: −0.172 to −0.029, *p* = 0.006). In an exploratory subgroup analysis, participants with higher DN4 scores (≥ 4) had a higher DTI-ALPS index than those with lower DN4 scores (<4) (1.270 vs. 1.108, *p* = 0.023). In patients with PHN, the DTI-ALPS index was negatively correlated with age (*r* = −0.397, *p* = 0.009) and positively correlated with DN4 score (*r* = 0.441, *p* = 0.003).

**Conclusion:**

Patients with PHN demonstrate altered perivascular-aligned water diffusivity as reflected by the DTI-ALPS index. These findings may reflect alterations in perivascular-aligned diffusivity, an indirect proxy related to glymphatic-associated processes. Further studies are warranted to clarify the relationship between neuropathic pain and glymphatic-related processes.

## 1 Introduction

Postherpetic neuralgia (PHN) is among the most challenging forms of neuropathic pain, affecting 10–20% of patients after herpes zoster (Johnson and Rice, 2014; Kawai et al., 2014). It is characterized by severe neuropathic pain persisting for more than 3 months after the rash resolves, substantially impairing quality of life and imposing a notable healthcare burden (Hadley et al., 2016; Jeon, 2015). The pathogenesis of PHN involves a complex interplay between peripheral and central mechanisms. Reactivation of latent varicella-zoster virus in sensory ganglia triggers a cascade of neuroinflammatory events, including the release of pro-inflammatory cytokines, activation of glial cells, and disruption of the blood-nerve barrier (Sampathkumar et al., 2009; Shen and Lin, 2025). These events result in direct nerve injury and central sensitization, altering central pain processing in the brain (Jaffal, 2025; Lacsa, 2026). Neuroimaging studies further suggest widespread central nervous system reorganization in patients with PHN, including altered brain network topology and abnormal structure–function coupling at the modular level (e.g., Default Mode, Salience, and Visual Networks) (Li et al., 2025).

Recent advances in neuroimaging have enabled non-invasive assessment of brain fluid dynamics and proxies related to glymphatic-associated processes (Iliff et al., 2012; Taoka et al., 2017; Park et al., 2025). Within brain fluid–solute physiology, cerebrospinal fluid (CSF)–interstitial fluid (ISF) exchange along perivascular pathways—conceptualized within the glymphatic model—supports transport and clearance of metabolites such as *β*-amyloid and inflammatory mediators (Jessen et al., 2015; Xie et al., 2013). Two-photon imaging studies have established para-arterial CSF influx and paravenous efflux facilitated by astrocytic aquaporin-4 (AQP4), providing a mechanistic basis for fluid–solute homeostasis (Iliff et al., 2012). Glymphatic system function is modulated by arousal state and neuromodulatory tone: sleep augments convective exchange and accelerates metabolite clearance, whereas wakeful norepinephrine activity restricts it (Xie et al., 2013). Complementing intraparenchymal routes, meningeal lymphatic vessels (MLVs) connect dural sinuses to deep cervical lymph nodes, providing extracranial efflux and linking fluid transport to immune surveillance (Louveau et al., 2015; Aspelund et al., 2015).

Diffusion tensor image analysis along the perivascular space (DTI-ALPS) method is a practical, non-invasive magnetic resonance imaging (MRI) method that samples directional diffusivities in white matter adjacent to deep medullary veins near the lateral ventricles (Taoka et al., 2017). In this study, we use the DTI-ALPS index as a perivascular-aligned diffusivity proxy in deep white matter (Taoka et al., 2017; Ringstad, 2024). Because the DTI-ALPS index is measured in supratentorial white matter, it yields a site-agnostic central readout applicable across cranial and non-cranial dermatomal involvement in patients with PHN (Taoka et al., 2017; Ozsahin et al., 2024). Notably, diffusion-MRI proxies linked to glymphatic transport exhibit age dependence and minimal or uncertain diurnal variability at current sensitivity in awake participants (Han et al., 2023). Measuring a perivascular-aligned diffusion proxy in patients with PHN is biologically reasonable on three grounds: (i) diffusion-based proxies linked to glymphatic transport exhibit age dependence and, in awake participants, minimal or uncertain time-of-day effects at current MRI sensitivity (Han et al., 2023); (ii) DTI-ALPS samples supratentorial deep white matter irrespective of peripheral lesion site, providing a site-agnostic central readout (Taoka et al., 2017; Ringstad, 2024); and (iii) sleep–pain reciprocity and AQP4-dependent astroglial processes suggest a plausible interface between perivascular water mobility and neuropathic symptoms, as supported by a glymphatic–pain perspective (Xie et al., 2013; Finan et al., 2013; Kourbanova et al., 2022; Lu et al., 2020; Guo et al., 2022; Goldman et al., 2020). However, the DTI-ALPS index should be interpreted as an indirect proxy rather than a direct measure of glymphatic function.

We hypothesized that patients with PHN had a lower DTI-ALPS index than age- and sex-matched healthy controls, and examined whether the DTI-ALPS index varies with clinical phenotype—neuropathic symptom burden (Douleur Neuropathique en 4, DN4), pain intensity (Numeric Rating Scale, NRS), or functional interference (Brief Pain Inventory, BPI). While the DTI-ALPS index provides a non-invasive proxy for perivascular function, it should be interpreted as an indirect marker rather than a direct measure of glymphatic clearance. We *a priori* interpret the DTI-ALPS index conservatively as a white-matter–weighted diffusion ratio reflecting composite effects of perivascular microstructure and fluid dynamics (Taoka et al., 2017; Ringstad, 2024).

## 2 Methods

**Figure 1:**
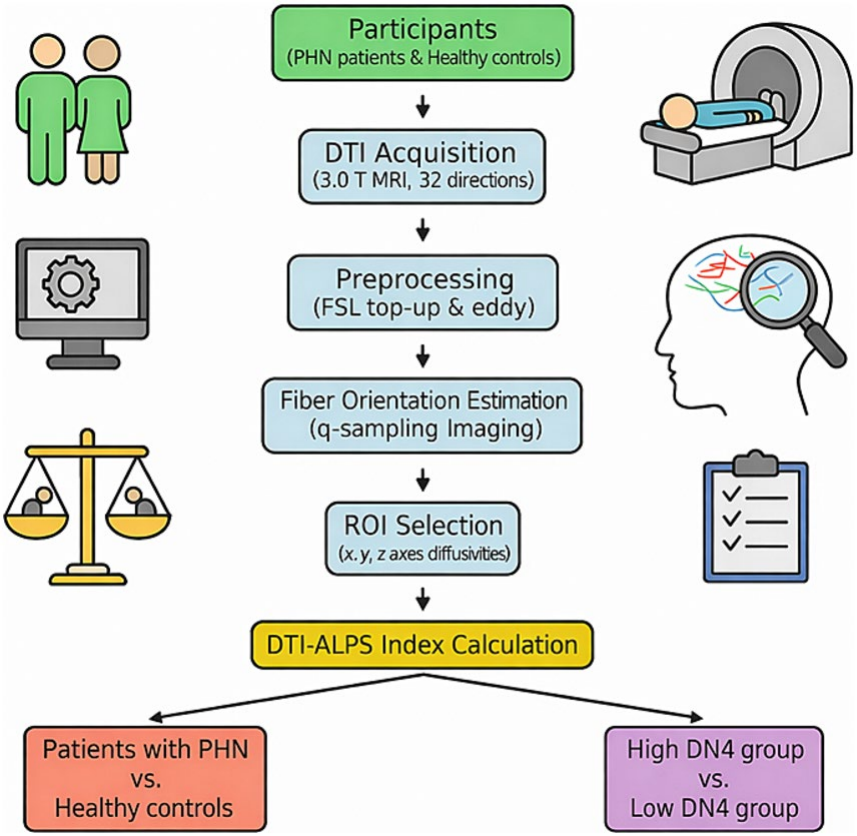
The process for this study. The diagram illustrates the overall procedure of this study. Participants included patients with PHN and age- and sex-matched healthy controls. All subjects underwent DTI acquisition using a 3.0 T MRI scanner. The DTI-ALPS index was calculated through image preprocessing, ROI-based analysis, and formula-based computation to assess perivascular-aligned water diffusivity. Clinical assessments (e.g., DN4, NRS, BPI) were obtained for patients with PHN. Finally, statistical analyses were performed to compare groups and evaluate correlations between the DTI-ALPS index and clinical characteristics. PHN, postherpetic neuralgia; DTI-ALPS, diffusion tensor image analysis along the perivascular space; DN4, Douleur neuropathique en 4 uestions; NRS, numeric rating scale; BPI, Brief Pain Inventory.

Figure 1 shows the process for this study.

### 2.1 Participants: patients with PHN and healthy controls

A total of 42 individuals with PHN were enrolled from the pain clinic at a university-affiliated hospital. PHN was diagnosed when patients experienced ongoing pain in a dermatomal pattern linked to a previous herpes zoster rash, with pain persisting for more than 3 months after the rash onset. Eligible participants were 18 years or older and able to provide informed consent. Exclusion criteria were: (1) a history of major neurological or psychiatric disorders (e.g., stroke, multiple sclerosis, or brain tumor), (2) uncontrolled systemic diseases, (3) contraindications to brain MRI scan, and (4) other chronic pain conditions unrelated to herpes zoster. Additionally, 41 healthy control participants, frequency-matched by age and sex, were recruited from the local community and confirmed to have no history of neuropathic pain or significant neurological disorders. All participants, patients with PHN and healthy controls, underwent structural MRI, and scans were reviewed to exclude major structural abnormalities, including silent lacunar infarctions, tumors, or other major abnormalities. However, age-related changes such as white matter hyperintensities or mild microangiopathy were not used as exclusion criteria. For PHN patients, symptom severity was evaluated using the DN4 score, a validated tool for assessing neuropathic pain that includes 10 items covering sensory descriptors and clinical examination findings. Furthermore, the following scales were used: (1) NRS for current pain intensity, and (2) BPI to assess pain-related functional interference. Pain intensity was measured on a 0–100 NRS to provide greater precision in discrimination (0 = no pain; 100 = worst imaginable pain).

### 2.2 DN4 assessment

The DN4 questionnaire comprises 10 items grouped into four sections: seven symptom descriptors (burning, painful cold, electric shocks, tingling, pins-and-needles, numbness, and itching) and three bedside signs (decreased light touch, decreased pinprick, and dynamic mechanical allodynia assessed with a soft brush). Each item is scored 0/1 (absent/present), yielding a total score of 0–10. For analyses, participants were dichotomized as DN4 screen-positive (≥4) versus screen-negative (<4), a threshold validated to indicate a high probability of neuropathic pain. Bedside testing was performed using a calibrated monofilament for light touch, a safety pin for pinprick, and a standardized soft brush for mechanical allodynia.

### 2.3 DTI acquisition

Both patients with PHN and healthy controls underwent DTI using a 3.0 T MRI scanner equipped with a 32-channel head coil. The DTI was performed with spin-echo single-shot echo-planar pulse sequences in a total of 32 different diffusion directions (TR/TE = 8620/85 ms, FA = 90°, slice thickness = 2.25 mm, acquisition matrix = 120 × 120, field of view = 240 × 240 mm^2^, and *b*-value = 1,000 s/mm^2^). The three-dimensional T1-weighted images were scanned (inversion time, 1,300 ms; repetition time/echo time, 8.6/3.96 ms; flip angle, 8^°^; and isotropic voxel size, 1 mm^3^).

### 2.4 DTI-ALPS index calculation

**Figure 2:**
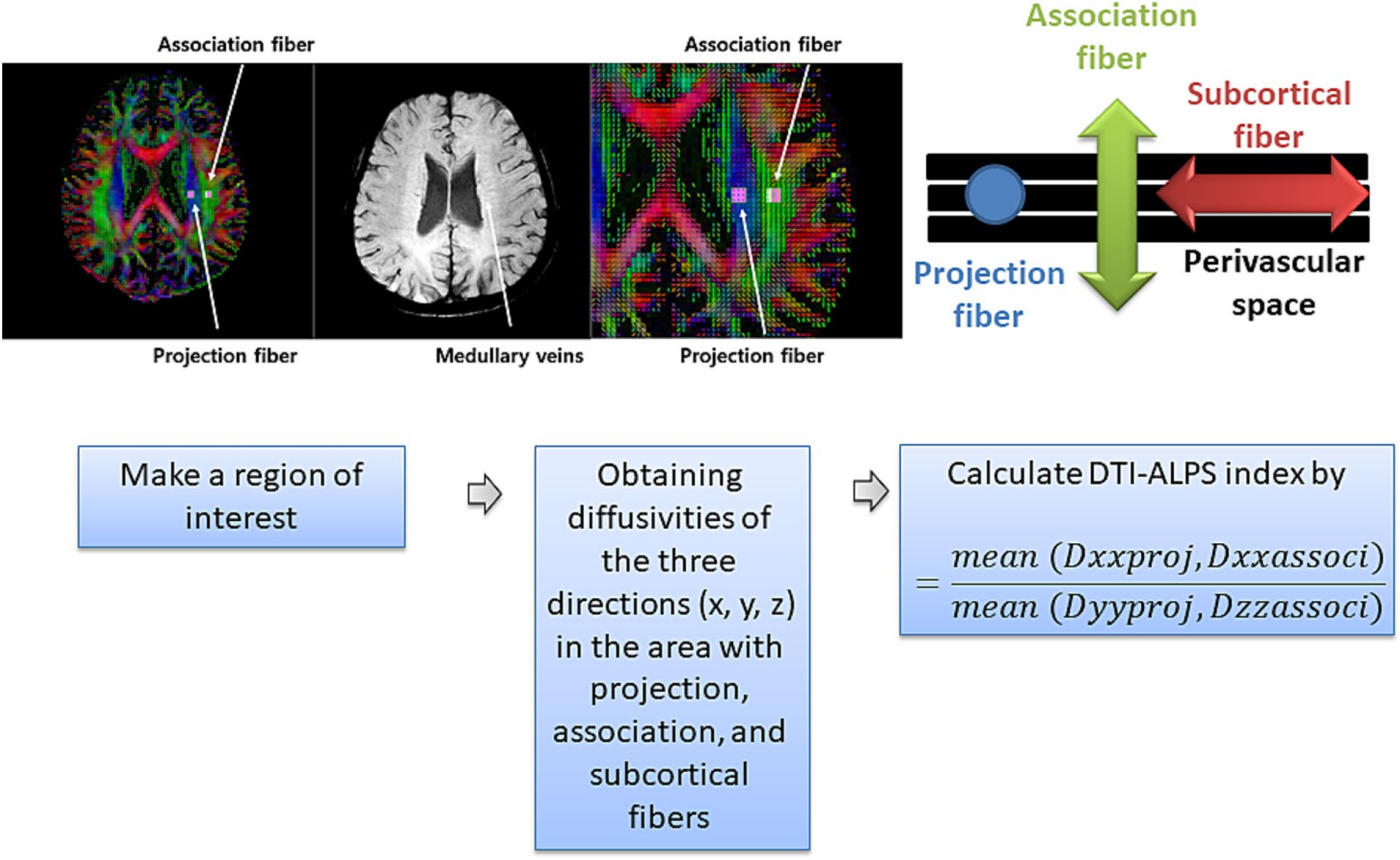
Schematic illustration of region-of-interest (ROI) placement and calculation of the DTI-ALPS index. ROIs were placed in areas containing projection, association, and subcortical fibers adjacent to the perivascular space. Diffusivities along the three orthogonal directions (*x, y*, and *z* axes) were obtained from each ROI. The DTI-ALPS index was calculated based on diffusivity components parallel and perpendicular to the perivascular space to reflect perivascular-aligned water diffusivity. Adapted from Lee DA et al. Epilepsia Open. 2022;7(2):306–314 under CC license.

The DTI-ALPS index was calculated as an indirect proxy of perivascular-aligned diffusivity related to glymphatic-associated processes in patients with PHN and healthy controls using DSI Studio. Quality control of diffusion data was performed prior to analysis. Raw NIFTI files were reviewed to confirm image integrity, including image dimensions, spatial resolution, and orientation matrices. Following preprocessing, all datasets were visually inspected for motion-related artifacts, signal dropout, and residual distortion. All scans were deemed to be of sufficient quality, and no datasets were excluded. Preprocessing was conducted using FSL’s top-up and eddy tools to correct for susceptibility-induced distortions and eddy-current artifacts. The source DTI images were then processed in DSI Studio. A background mask was created to remove non-brain regions and improve reconstruction efficiency. Thresholding, smoothing, and defragmentation were applied. A generalized q-sampling imaging method was subsequently used to generate a single fiber orientation per voxel and derive associated anisotropy and diffusivity measures. A region of interest (ROI) was manually delineated, from which fiber orientation and diffusivities were extracted along the x, y, and z axes as voxel values within the ROI. Finally, the DTI-ALPS index was computed using the following formula (Figure 2) (Taoka et al., 2017):

$$\mathrm{DTI}-\text{ALPS index}=\frac{\text{mean}\;(\text{Dxxproj},\text{Dxxassoci})}{\text{mean}\;(\text{Dyyproj},\text{Dzzassoci})}$$

Where, Dxxproj is the diffusivity along the x-axis in the projection fiber, Dxxassoci is the diffusivity along the x-axis in the association fiber, Dyyproj is the diffusivity along the y-axis in the projection fiber, and Dzzassoci is the diffusivity along the z-axis in the association fiber.

To assess inter-rater reliability of ROI placement, two independent raters performed ROI delineation in a subset of subjects. Inter-rater reliability was evaluated using the intraclass correlation coefficient (ICC) based on a two-way random-effects model with absolute agreement.

### 2.5 White matter hypointensity volume analysis

To segment white matter hypointensity from T1-weighted images and acquire white matter hypointensity volume, we used WMH-SynthSeg, which provides segmentation for white matter hypointensity from scans of any resolution and contrast without retraining, available as module in the development version of FreeSurfer. The white matter hypointensity volume was corrected with total intracranial volume (white matter hypointensity volume/total intracranial volume × 100).

### 2.6 Correlation analysis

We conducted a correlation analysis between the measures of the DTI-ALPS index and clinical characteristics, including age, DN4, NRS, and BPI score in patients with PHN.

### 2.7 Statistical analysis

The categorical variables were analyzed using the Chi-square test or Fisher’s exact test, whereas continuous variables were tested with the independent samples *t*-test or Mann–Whitney U test, depending on data normality. The correlation analysis was conducted with Pearson’s test. To account for the potential influence of age and white matter hypointensity volume on DTI-ALPS index, an analysis of covariance (ANCOVA) was performed with DTI-ALPS index as the dependent variable, group (PHN vs. healthy controls; High DN4 group vs. Low DN4 group) as the fixed factor, and age and white matter hypointensity volume as covariates. This analysis was conducted to determine whether the between-group difference in DTI-ALPS index remained significant after adjusting for differences in age and white matter hypointensity volume. Assumptions for ANCOVA were evaluated prior to analysis. Normality of residuals was confirmed using the Shapiro–Wilk test, and homogeneity of regression slopes was verified. Homogeneity of variances was assessed using Levene’s test. The DN4 subgroup analysis was conducted as an exploratory analysis and was not part of the primary hypothesis testing framework. Multiple linear regression analyses were performed to evaluate factors associated with the DTI-ALPS index. In the overall cohort, PHN status was included as the primary independent variable, with adjustment for age and white matter hypointensity volume. In addition, within the PHN group, a similar model was applied to assess the association between DN4 subgroup (high vs. low) and the DTI-ALPS index, adjusting for the same covariates. All statistical analyses were performed using MedCalc® Statistical Software version 23.2.7 (MedCalc Software Ltd., Ostend, Belgium; https://www.medcalc.org; 2025). A *p*-value <0.05 was considered statistically significant. To account for multiple comparisons, Bonferroni correction was applied to the analyses of directional diffusivity measures (Dxx, Dyy, Dzz across projection, association, and subcortical fibers). A corrected significance threshold of *p* < 0.0056 (0.05/9) was considered statistically significant. Bonferroni correction was applied only to the analyses of directional diffusivity measures. Other analyses, including subgroup and correlation analyses, were considered exploratory and were not adjusted for multiple comparisons. In addition to *p*-values, effect sizes were calculated to assess the magnitude of group differences. Cohen’s d was computed for comparisons between groups, and 95% confidence intervals were estimated to quantify the precision of the observed effects. Categorical variables were expressed as frequency with percentage, and continuous variables were expressed as mean ± standard deviation (SD) or median with interquartile range (IQR), according to the data distribution.

## 3 Results

### 3.1 Clinical characteristics

Table 1 shows the clinical characteristics of all the participants. No significant differences were observed in mean age or sex distribution between patients with PHN and healthy controls. Among the 42 patients with PHN, 34 (81.0%) were assigned to the high DN4 group and 8 (19.0%) to the low DN4 group. Similarly, no significant differences were found in mean age or sex distribution between these two subgroups. In addition, pain intensity measured by the NRS and functional interference assessed by the BPI did not differ significantly between the high and low DN4 groups. The proportion of patients using GABAergic drugs, antidepressants, opioids, or NSAIDs was comparable between groups, with no statistically significant differences in medication category.

**Table 1:** Clinical characteristics of patients with postherpetic neuralgia and healthy controls.

	Patients with PHN (*N* = 42)	Healthy controls (*N* = 41)	*p*-value
Mean Age ±SD, years	70.4 ± 10.3	69.3 ± 6.5	0.595
Male, *n* (%)	32 (76.1)	32 (78.0)	0.841
Disease duration, months (interquartile range)	19.1 (5.2–53.1)		
Median NRS (interquartile range)	45 (30–60)		
Right/Left side of involved nerve area, *n* (%)	18 (42.8) / 24 (57.2)		
Involved nerve area			
Cranial area, *n* (%)	15 (35.7)		
Cervical area, *n* (%)	5 (11.9)		
Thoracic area, *n* (%)	21 (50.0)		
Lumbar area, *n* (%)	1 (2.4)		
Median DN4 (interquartile range)	5 (4–6)		
Median BPI (interquartile range)	25 (17–33)		
Medications			
GABAergic drug, *n* (%)	42 (100.0)		
Antidepressant, *n* (%)	19 (45.2)		
Opioid, *n* (%)	23 (54.8)		
NSAID, *n* (%)	9 (21.4)		

	High DN4 group (*N* = 34)	Low DN4 group (*N* = 8)	*p*-value
Mean Age ±SD, years	70.6 ± 10.4	69.4 ± 10.2	0.758
Male, *n* (%)	25 (73.5)	7 (87.5)	0.654
Disease duration, months (interquartile range)	19.1 (5.2–61.5)	25.2 (5.6–41.6)	0.961
Median NRS (interquartile range)	45 (30–60)	45 (30–55)	0.807
Right side, *n* (%)	15 (44.1)	3 (37.6)	1.000
Median DN4 (interquartile range)	6 (5–6)	2 (1–3)	< 0.001
Median BPI (interquartile range)	26 (19–34)	21 (13–28)	0.188
Medications			0.836
GABAergic drug, *n* (%)	34 (100.0)	8 (100.0)	
Antidepressant, *n* (%)	17 (50.0)	2 (25.0)	
Opioid, *n* (%)	19 (55.9)	4 (50.0)	
NSAID, *n* (%)	7 (20.6)	2 (25.0)	

PHN, postherpetic neuralgia; SD, standard deviation; NRS, numeric rating scale; DN4, Douleur neuropathique en 4 questions; BPI, brief pain inventory; GABA, gamma-aminobutyric acid; NSAID, non-steroidal anti-inflammatory drug.

### 3.2 DTI-ALPS index

Inter-rater reliability for ROI placement was high, with an ICC of 0.87 (95% confidence interval: 0.78–0.93), indicating good agreement between raters.

**Figure 3:**
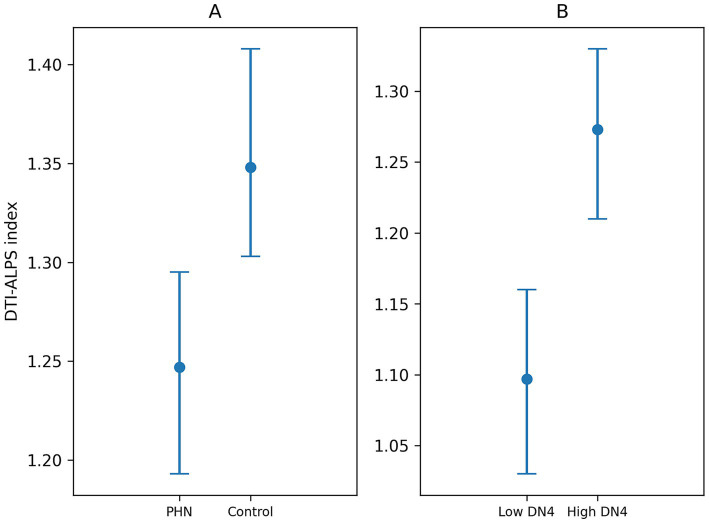
Adjusted group comparisons of the DTI-ALPS index. Estimated marginal means of the DTI-ALPS index for patients with PHN and healthy controls, derived from ANCOVA adjusted for age and white matter hypointensity volume **(A)**. Estimated marginal means from an exploratory subgroup comparison according to DN4 score (DN4 < 4 vs. DN4 ≥ 4), derived from ANCOVA adjusted for age and white matter hypointensity volume **(B)**. Error bars indicate 95% confidence intervals. PHN, postherpetic neuralgia; DTI-ALPS, diffusion tensor image analysis along the perivascular space; DN4, Douleur Neuropathique en 4 questions.

Table 2 shows the differences in diffusivities and the DTI-ALPS index between patients with PHN and healthy controls. Diffusivities along the y-axis in both the projection and association fibers were significantly higher in patients with PHN than in healthy controls (0.57 × 10^−3^ vs. 0.51 × 10^−3^, *p* = 0.005; 1.13 × 10^−3^ vs. 1.07 × 10^−3^, *p* = 0.005; respectively), and these differences remained significant after Bonferroni correction. In contrast, diffusivities along the z-axis in the association and subcortical fibers were also higher in patients with PHN than in healthy controls (0.44 × 10^−3^ vs. 0.40 × 10^−3^, *p* = 0.014; 0.66 × 10^−3^ vs. 0.59 × 10^−3^, *p* = 0.045; respectively), but these differences did not remain statistically significant after correction for multiple comparisons. Importantly, the DTI-ALPS index was significantly lower in patients with PHN compared with healthy controls (1.239 vs. 1.356, *p* = 0.001). In the adjusted model using ANCOVA, group remained a significant predictor of the DTI-ALPS index (*F* = 7.858, *p* = 0.006), after controlling for age and white matter hypointensity volume. Age was also significantly associated with the DTI-ALPS index (*p* = 0.005), whereas white matter hypointensity volume was not (*p* = 0.444). The overall model explained approximately 23.4% of the variance (*R*^2^ = 0.234). Assumption testing indicated that the homogeneity of regression slopes was satisfied (*p* = 0.687), supporting the validity of the ANCOVA model. Residuals were normally distributed (Shapiro–Wilk *p* = 0.529). Although Levene’s test indicated a mild deviation from homogeneity of variance (*p* = 0.032), ANCOVA is considered robust to moderate violations, and the main findings were consistent. Estimated marginal means were 1.247 in the PHN group and 1.348 in the control group (mean difference = −0.100, 95% CI: −0.172 to −0.029) (Figure 3A). In the multiple linear regression model with the DTI-ALPS index as the dependent variable, PHN status remained significantly associated with the DTI-ALPS index after adjustment for age and white matter hypointensity volume (*β* = −0.092, SE = 0.036, 95% CI: −0.165 to −0.020, *p* = 0.012). Age was also significantly associated with the DTI-ALPS index (*β* = −0.003, SE = 0.001, 95% CI: −0.007 to −0.000, *p* = 0.014), whereas white matter hypointensity volume was not (*β* = −0.109, SE = 0.098, 95% CI: −0.304 to 0.085, *p* = 0.267).

**Table 2:** Differences of the diffusivities and DTI-ALPS index between patients with postherpetic neuralgia and healthy controls.

	Patients with PHN (*N* = 42)	Healthy controls (*N* = 41)	*p* value	95% CI	Cohen’s d
Projection fiber					
Dxx	0.582 ± 0.092	0.564 ± 0.061	0.326	−0.015 to 0.051	0.23
Dyy	0.570 ± 0.103	0.511 ± 0.074	0.005	0.023 to 0.095	0.66
Dzz	1.090 ± 0.111	1.020 ± 0.126	0.079	−0.009 to 0.149	0.60
Association fiber					
Dxx	0.664 ± 0.108	0.670 ± 0.081	0.774	−0.046 to 0.034	−0.06
Dyy	1.130 ± 0.091	1.070 ± 0.088	0.005	0.020 to 0.100	0.67
Dzz	0.449 ± 0.082	0.408 ± 0.069	0.014	0.007 to 0.075	0.67
Subcortical fiber					
Dxx	1.120 ± 0.101	1.090 ± 0.110	0.188	−0.015 to 0.075	0.29
Dyy	0.710 ± 0.168	0.673 ± 0.144	0.280	−0.030 to 0.104	0.24
Dzz	0.666 ± 0.173	0.596 ± 0.140	0.045	0.001 to 0.139	0.45
DTI-ALPS index	1.239 ± 0.184	1.356 ± 0.140	0.001	−0.188 to −0.046	−0.71

	High DN4 group (*N* = 34)	Low DN4 group (*N* = 8)	*p* value	95% CI	Cohen’s d
Projection fiber					
Dxx	0.587 ± 0.096	0.559 ± 0.079	0.461	−0.055 to 0.111	0.30
Dyy	0.561 ± 0.102	0.608 ± 0.108	0.258	−0.142 to 0.048	−0.45
Dzz	1.090 ± 0.114	1.090 ± 0.102	0.982	−0.092 to 0.092	0.00
Association fiber					
Dxx	0.675 ± 0.110	0.616 ± 0.090	0.168	−0.030 to 0.148	0.57
Dyy	1.150 ± 0.085	1.060 ± 0.092	0.187	−0.015 to 0.195	1.01
Dzz	0.444 ± 0.080	0.472 ± 0.092	0.394	−0.116 to 0.060	−0.34
Subcortical fiber					
Dxx	1.120 ± 0.092	1.110 ± 0.142	0.908	−0.090 to 0.110	0.09
Dyy	0.698 ± 0.140	0.764 ± 0.262	0.322	−0.217 to 0.085	−0.42
Dzz	0.666 ± 0.161	0.665 ± 0.230	0.98	−0.169 to 0.171	0.01
DTI-ALPS index	1.270 ± 0.174	1.108 ± 0.180	0.023	0.006 to 0.318	0.93

(×10^−3^ mm^2^ / s).

Values are presented as mean ± standard deviation. Mean differences are presented with 95% confidence intervals (CI). Effect sizes are reported as Cohen’s d. Dxx, diffusivity along the x-axis; Dyy, diffusivity along the y-axis; Dzz, diffusivity along the z-axis; DTI-ALPS index, diffusion tensor image analysis along the perivascular space index; PHN, postherpetic neuralgia; SD, standard deviation; DN4, Douleur neuropathique en 4 questions.

In an exploratory subgroup analysis, the DTI-ALPS index and diffusivity measures were compared according to DN4 score. The directional diffusivity measures did not differ significantly between the high-DN4 and low-DN4 subgroups. The DTI-ALPS index was higher in the high-DN4 subgroup than in the low-DN4 subgroup (1.270 vs. 1.108, *p* = 0.023). After adjusting for age and white matter hypointensity volume using ANCOVA, the difference in the DTI-ALPS index between high and low DN4 subgroups remained statistically significant (*F* = 7.528, *p* = 0.009). Age was also significantly associated with the DTI-ALPS index (*F* = 5.947, *p* = 0.020), whereas white matter hypointensity volume was not (*F* = 0.189, *p* = 0.666). The overall model explained 29.7% of the variance (*R*^2^ = 0.297). Assumption testing supported the validity of the ANCOVA model. The homogeneity of regression slopes was satisfied (*p* = 0.329), and residuals were normally distributed (Shapiro–Wilk *p* = 0.344). Homogeneity of variance was also met (Levene’s test *p* = 0.177). Estimated marginal means were 1.273 in the high DN4 group and 1.097 in the low DN4 group. The adjusted mean difference was 0.176 (95% CI: 0.046 to 0.306) (Figure 3B). In a multiple linear regression model within the PHN group, DN4 subgroup (high vs. low) remained significantly associated with the DTI-ALPS index after adjustment for age and white matter hypointensity volume (*β* = −0.176, SE = 0.064, 95% CI: −0.306 to −0.046, *p* = 0.009). Age was also significantly associated with the DTI-ALPS index (*β* = −0.006, SE = 0.002, 95% CI: −0.012 to −0.001, *p* = 0.019), whereas white matter hypointensity volume was not (*β* = −0.061, SE = 0.142, 95% CI: −0.350 to 0.226, *p* = 0.666).

To examine the potential influence of medications, exploratory subgroup analyses were performed within the PHN group according to opioid and antidepressant use. No significant differences in DTI-ALPS index were observed between patients receiving opioids and those not receiving opioids (1.270 vs. 1.202, *p* = 0.245). Similarly, the DTI-ALPS index did not differ between patients treated with antidepressants and those not receiving antidepressants (1.254 vs. 1.228, *p* = 0.654), suggesting no clear evidence of a difference in DTI-ALPS index according to these medication classes in these exploratory comparisons. To further address potential medication confounding, a multivariable linear regression analysis was performed within the PHN group, including age, white matter hypointensity volume, opioid use, and antidepressant use as independent variables. In this model, neither opioid use (*p* = 0.369) nor antidepressant use (*p* = 0.949) was significantly associated with the DTI-ALPS index. Age remained significantly associated with the DTI-ALPS index (*β* = −0.007, *p* = 0.032), whereas white matter hypointensity volume was not (*p* = 0.928). The overall model was not statistically significant (*F* = 1.984, *p* = 0.117), and explained 17.7% of the variance (R^2^ = 0.177). Residuals were normally distributed (Shapiro–Wilk *p* = 0.404).

### 3.3 Correlations between measures of DTI-ALPS index and clinical characteristics

**Figure 4:**
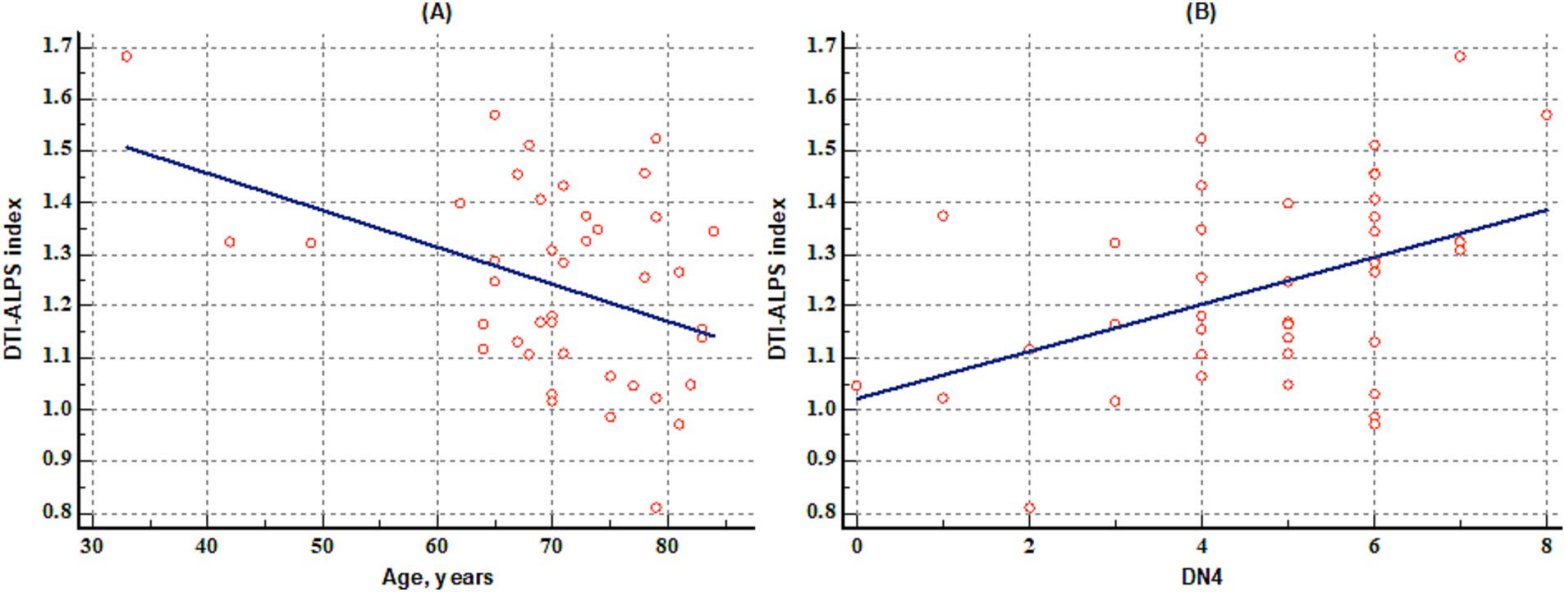
The results of the correlation analysis. The figures show that age is negatively correlated with diffusion tensor image analysis along the perivascular space (DTI-ALPS index) (*r* = −0.397, *p* = 0.009) **(A)**, and DN4 score is positively correlated with the DTI-ALPS index (*r* = 0.441, *p* = 0.003) **(B)** in patients with postherpetic neuralgia. DN4: Douleur Neuropathique en 4 Questions.

Among patients with PHN, the DTI-ALPS index showed a negative correlation with age (*r* = −0.397, *p* = 0.009) (Figure 4A) and a positive correlation with DN4 score (*r* = 0.441, *p* = 0.003) (Figure 4B). However, the DTI-ALPS index was not significantly associated with NRS (*r* = 0.221, *p* = 0.158) or BPI score (*r* = 0.096, *p* = 0.541).

### 3.4 Group difference in white matter hypointensity volumes

The corrected white matter hypointensity volumes were higher in patients with PHN than in the healthy controls (0.218 ± 0.206 vs. 0.094 ± 0.164%, *p* = 0.003).

## 4 Discussion

The present study demonstrates a group-level reduction in the DTI-ALPS index among patients with PHN compared with healthy controls. The observed group difference remained statistically significant after adjustment for age and white matter hypointensity volume, suggesting that it is not fully explained by age-related or structural white matter changes. Within the PHN cohort, age was negatively correlated with the DTI-ALPS index, whereas neuropathic symptom burden (DN4 score) showed a positive correlation. In contrast, pain intensity (NRS) and functional interference (BPI) were not significantly associated. Taken together, these findings suggest altered perivascular-aligned water diffusivity in PHN, with phenotype-linked variation more closely related to neuropathic characteristics than to current pain intensity or functional impairment.

Several non-exclusive mechanisms may contribute to lower perivascular-aligned diffusivity in PHN. Chronic sleep disruption and elevated arousal/noradrenergic tone can suppress convective CSF–ISF exchange, consistent with sleep-linked augmentation and wake-linked suppression of glymphatic-associated transport. Neuroinflammatory remodeling of astroglia, including AQP4 depolarization at astrocytic endfeet, may further impair para-arterial influx and paravenous efflux. Additionally, pain-related autonomic dysregulation, compounded by age-related vascular stiffness, could narrow or alter perivascular conduits sampled by the DTI-ALPS index. Together, these factors provide a biologically coherent, albeit non-causal, link between PHN pathophysiology and lower DTI-ALPS index values (Xie et al., 2013; Iliff et al., 2013; Nelson-Maney et al., 2024; Wu et al., 2025). In addition, meningeal-lymphatic signaling (e.g., calcitonin gene-related peptide [CGRP]-linked pathways) could secondarily influence CSF efflux and venous drainage in susceptible phenotypes (Nelson-Maney et al., 2024).

Within the fluid–glia–vascular framework, sleep enhances convective CSF–ISF exchange and facilitates metabolite mobilization (Xie et al., 2013). AQP4 supports perivascular water transport through astrocytic endfeet (Iliff et al., 2012), while MLVs connect intracranial solute traffic to extracranial immune pathways (Louveau et al., 2015; Aspelund et al., 2015). Clinically, patients with PHN frequently experience significant insomnia (Lee et al., 2016), and experimental migraine models implicate CGRP-driven meningeal-lymphatic signaling in the modulation of CSF efflux and nociceptive processing (Nelson-Maney et al., 2024). Together with reports linking sleep disruption to heightened pain sensitivity (Finan et al., 2013; Kourbanova et al., 2022) and AQP4-dependent astroglial processes to neuropathic pain phenotypes (Lu et al., 2020; Guo et al., 2022), these observations outline a hypothesis-generating pathway in which arousal state and glial-lymphatic interactions may modulate pain. Our findings are consistent with this pathway: lower DTI-ALPS values in patients with PHN may reflect differences in perivascular-aligned diffusivity beyond the effects of healthy aging, and the positive correlation with DN4 scores suggests that the DTI-ALPS index is sensitive to phenotype-related variation. Definitive validation will require multimodal imaging studies with strict control of arousal state, time of day, and direct lymphatic readouts.

Neuroimaging studies in patients with PHN have reported white-matter microstructural alterations and functional network differences (Jiang et al., 2022; Cao et al., 2017; Li et al., 2022). The present finding of reduced DTI-ALPS index is consistent with these reports, suggesting that perivascular-aligned diffusivity may also be affected alongside other central alterations. Importantly, diffusion-based measures linked to glymphatic transport are age-dependent and, in awake participants, exhibit minimal or uncertain time-of-day effects at current MRI sensitivity (Han et al., 2023). Consistent with this, animal studies indicate that aging-related AQP4 depolarization impairs paravascular clearance (Kress et al., 2014), and emerging 24-h human scanning studies are exploring potential diurnal modulation of DTI-ALPS index, although existing designs are heterogeneous and reported effects remain small (Ko et al., 2025).

We examined the DN4 because it captures trait-like neuropathic qualities (e.g., allodynia, paresthesia) that align more closely with central sensitization and sensory profile rather than transient pain intensity. Accordingly, a positive association between DN4 score and DTI-ALPS index may suggest that perivascular-aligned diffusivity may track phenotype-linked neuropathic features (Iliff et al., 2012; Xie et al., 2013; Nelson-Maney et al., 2024; Zeppenfeld et al., 2017). The paradoxical positive correlation between DN4 scores and the DTI-ALPS index warrants careful interpretation. The direction of this association appears counterintuitive relative to the primary group comparison, further supporting cautious interpretation. This pattern may also reflect statistical instability due to the small subgroup size rather than a robust biological effect. Given the small and unbalanced subgroup sizes, particularly the limited number of participants in the low-DN4 group (*n* = 8), this finding should be considered exploratory. The relatively large effect size observed in this subgroup analysis may be influenced by sampling variability and potential inflation of effect size estimates. This counterintuitive pattern suggests that severe neuropathic symptoms may not solely reflect impaired glymphatic-related processes. Rather, relatively preserved perivascular-aligned diffusivity in patients with higher DN4 scores may reflect heterogeneity in neuropathic phenotypes or state-dependent adaptations; however, these interpretations remain speculative (Goldman et al., 2020; Zhang et al., 2022). Accordingly, these associations should be interpreted cautiously and require validation in larger, balanced cohorts with appropriate adjustment for potential confounders, including age, vascular and white-matter burden, medication use, lesion level, and potential nonlinear effects. While this observation may generate hypotheses regarding phenotype-linked variation in perivascular-aligned diffusivity, its clinical implications remain uncertain and should not be overinterpreted at the present stage (Taoka and Naganawa, 2020). This pattern is broadly compatible with models in which astroglial/AQP4-dependent processes and sleep–pain reciprocity shape central phenotype expression, while acknowledging potential microstructural and vascular confounders within the DTI-ALPS index (Wu et al., 2025). Therefore, this finding should be regarded as hypothesis-generating and not confirmatory.

Methodologically, the DTI-ALPS method is an indirect, white-matter–weighted surrogate that depends on fiber geometry, ROI placement, and orientation, and remains susceptible to crossing-fiber effects (Ringstad, 2024; Georgiopoulos et al., 2024; Schilling et al., 2025). We mitigated these factors by employing standardized ventricular-level ROIs, implementing rater blinding with reliability assessment, enforcing motion/registration quality control, and applying *a priori* constraints to minimize crossing-fiber contamination (Georgiopoulos et al., 2024; Schilling et al., 2025). Beyond our protocol, cross-vendor and test–retest studies demonstrate robust inter-scanner, inter-rater, and test–retest reliability for the DTI-ALPS index (Tatekawa et al., 2023). Moreover, population-based analyses reveal strong associations of the DTI-ALPS index with age, vascular risk, and white-matter hyperintensity burden, but comparatively weak associations with Alzheimer’s biomarkers (Satpathi et al., 2025), reinforcing a cautious, milieu-oriented interpretation rather than a disease-specific inference. To improve interpretability, we reported effect sizes and 95% confidence intervals, applied a covariate-adjusted model for the primary group comparison, and used Bonferroni correction for secondary directional diffusivity analyses.

At present, the DTI-ALPS method should be regarded as a non-invasive, site-agnostic central marker that reflects perivascular-aligned diffusion but is not disease-specific. In patients with PHN, such a marker could be valuable for stratifying neuropathic phenotypes (e.g., DN4-linked features) and tracking interventions that plausibly engage the sleep–glia–vascular axis, such as sleep optimization, autonomic modulation, or astroglial-targeted approaches, while maintaining appropriate mechanistic caution. Clinically, the DTI-ALPS index could serve as a potential biomarker for phenotyping patients with PHN based on neuropathic symptom severity, monitoring treatment response to sleep-targeted interventions such as sleep hygiene optimization (Finan et al., 2013) or circadian rhythm modulation (Kourbanova et al., 2022), and identifying patients at risk of prolonged or treatment-resistant neuropathic pain. The positive correlation between the DTI-ALPS index and DN4 scores suggests that patients with more severe neuropathic symptoms may exhibit relatively preserved perivascular-aligned diffusivity (an indirect proxy related to glymphatic-associated processes), which could inform individualized treatment approaches while avoiding mechanistic overreach. Because the DTI-ALPS index is straightforward to compute from widely available diffusion datasets (Taoka et al., 2017) and demonstrates robust cross-vendor and test–retest characteristics (Liu et al., 2024), it may facilitate multi-site harmonization and prospective replication, including state-controlled designs (pre−/post-sleep or nap paradigms) and multimodal readouts (e.g., combining diffusion-based proxies with venous-space mapping or lymphatic imaging) (Iliff et al., 2012; Xie et al., 2013; Louveau et al., 2015; Aspelund et al., 2015; Han et al., 2023; Goldman et al., 2020; Nelson-Maney et al., 2024; Ko et al., 2025; Georgiopoulos et al., 2024). We emphasize that group contrast should always be accompanied by effect sizes, confidence intervals, covariate-adjusted models, and appropriate control for multiple comparisons to enhance interpretability and reproducibility.

Several limitations should be acknowledged. First, the cross-sectional design limits causal inference regarding the relationship between DTI-ALPS alterations, as an indirect proxy related to glymphatic-associated processes, and PHN pathophysiology. Longitudinal studies are needed to determine whether DTI-ALPS index alterations precede, accompany, or follow PHN onset and progression. Second, the DTI-ALPS index represents an indirect marker of perivascular function rather than a direct measure of glymphatic clearance. While correlations with tracer-based glymphatic imaging have been demonstrated (Xie et al., 2013; Louveau et al., 2015), the DTI-ALPS index likely reflects composite effects of tissue microstructure, fiber geometry, and fluid dynamics that may be confounded by white matter pathology or individual anatomical variability. Third, our study has methodological limitations, including single-center recruitment, a relatively small sample size (particularly the low DN4 subgroup, n = 8), and a lack of systematic circadian control for potential time-of-day variation in glymphatic activity. Additionally, although we explored potential medication confounding using subgroup comparisons and a multivariable regression model, we did not systematically control for medication dose, duration, or cumulative sedative burden, and residual confounding remains possible. Furthermore, the lack of a statistically significant association between medication use and the DTI-ALPS index should be interpreted with caution, as the study may have been underpowered to detect a potential effect of medications. Fourth, all scans were performed during wakefulness, despite evidence that glymphatic system activity shows pronounced sleep–wake differences (Jessen et al., 2015). Future studies should incorporate sleep-state imaging or, at a minimum, standardize circadian timing to better characterize physiologically relevant variation in DTI-ALPS-related perivascular fluid dynamics. Finally, validation in larger, multi-site cohorts with explicit mechanistic endpoints (e.g., CSF biomarkers, sleep studies, or direct glymphatic imaging) will be essential to establish the DTI-ALPS index as a clinically meaningful biomarker in chronic pain conditions.

## 5 Conclusion

These findings indicate that PHN is associated with altered perivascular-aligned diffusivity in deep white matter, as reflected by a lower DTI-ALPS index relative to age- and sex-matched healthy controls. Within the PHN cohort, the DTI-ALPS index showed differential associations with key clinical features, including declining with patient age but increasing with greater neuropathic symptom severity.

## Data Availability

The raw data supporting the conclusions of this article will be made available by the authors, without undue reservation.
